# The association between hydration state and the metabolism of phospholipids and amino acids among young adults: a metabolomic analysis

**DOI:** 10.1016/j.cdnut.2024.102087

**Published:** 2024-02-01

**Authors:** Yongwei Lin, Na Zhang, Jianfen Zhang, Junbo Lu, Shufang Liu, Guansheng Ma

**Affiliations:** 1 Department of Nutrition and Food Hygiene, School of Public Health, Peking University, Beijing, China; 2 Laboratory of Toxicological Research and Risk Assessment for Food Safety, Beijing, China; 3 School of Public Health, Hebei University Health Science Center, Baoding, China

**Keywords:** hydration state, urine, metabolites, metabolomics, phospholipid, amino acid

## Abstract

**Background:**

Water is vital for humans' survival and general health, which is involved in various metabolic activities.

**Objectives:**

The aim of this study was to investigate the variation in urine metabolome and associated metabolic pathways among people with different hydration states.

**Methods:**

A metabolomic analysis was conducted using 24-h urine samples collected during a cross-sectional study on fluid intake behavior from December 9 to 11, 2021, in Hebei, China. Subjects were divided into the optimal hydration (OH, ≤500 mOsm/kg, *n* = 21), middle hydration (500–800 mOsm/kg, *n* = 33), and hypohydration groups (HH, >800 mOsm/kg, *n* = 13) based on the 3-d average 24-h urine osmolality. Collected 24-h urine samples from 67 subjects (43 males and 34 females) were analyzed for urine metabolome using liquid chromatography-MS.

**Results:**

The untargeted metabolomic analysis yielded 1055 metabolites by peak intensities. Integrating the results of the orthogonal projections to latent structures discriminant analysis and fold change test, 115 differential metabolites between the OH and HH groups, including phospholipids (PLs) and lysophospholipids, were identified. Among the 115 metabolites identified as differential metabolites, 85 were recorded by the Human Metabolome Database and uploaded to the Kyoto Encyclopedia of Genes and Genomes databases for pathway analysis. Twenty-one metabolic pathways were recognized. Phenylalanine metabolism (0.50, *P* = 0.007), phenylalanine, tyrosine, and tryptophan biosynthesis (0.50, *P* = 0.051), glycerophospholipid metabolism (0.31, *P* < 0.001), sphingolipid metabolism (0.27, *P* = 0.029), and cysteine and methionine metabolism (0.10, *P* = 0.066) had the leading pathway impacts.

**Conclusions:**

We found variations in the urinary PLs and amino acids among subjects with different hydration states. Pathways associated with these differential metabolites could further impact various physiologic and pathologic functions. A more comprehensive and in-depth investigation of the physiologic and pathologic impact of the hydration state and the underlying mechanisms to elucidate and advocate optimal fluid intake habits is needed.

This trial was registered at Chinese Clinical Trial Registry as ChiCTR2100045268.

## Introduction

Water is the fundamental building material of the human body, distributed widely in cells, tissues, and organs, thereby serving various vital physiologic functions, including but not limited to maintaining the electrolyte balance, regulating the body temperature, and lubricating joints [1].

Water within the body usually maintains homeostasis, as the amount of water gain and loss remains roughly balanced under close monitoring and regulation of the central nervous system and multiple hormones. Arginine vasopressin (AVP), for instance, is the hormone that acts on the kidney to promote fluid reabsorption and retention. A subtle change as small as 1% in serum osmolality would activate the regulatory system, triggering physiologic changes that could further impact one’s metabolism and overall health [1].

Interruption in the homeostasis state could result in various adverse health outcomes and be fatal in rare cases. Dehydration, usually due to inadequate fluid intake or excessive loss of body fluid, could impede cognitive function. Conversely, overhydration, although rare, could result in water toxication, causing headaches, nausea, and memory loss [1].

In addition to the acute health impacts mentioned above, one’s hydration state and associated hormonal changes could lead to chronic health consequences. Long-term dehydration may impair kidney function and be associated with higher incidences of various chronic diseases, such as cardiovascular diseases and diabetes mellitus. Extensive research has revealed the role hydration and related hormones play in various metabolic conditions, specifically glycemic control and the onset of type II diabetes mellitus [2]. An elevated concentration of AVP and its surrogate biomarker, co-peptin, were associated with a higher incidence of cardiocerebral and metabolic diseases [[3], [4], [5]].

Increasing studies have also discovered and solidified the underlying physiologic basis of how the hydration state may alter one’s nutrient metabolism. Low osmolality, for instance, can lead to cell swelling and interfere with glycogenolysis and proteolysis in the liver. In contrast, cell shrinkage caused by high osmolality can promote glycogenolysis, glycolysis, and proteolysis [6]. In a study investigating the relationship between hydration biomarkers and energy metabolism, participants with less optimal hydration state associated with lower urine volume and higher urine nitrogen were found to have lower respiratory quotient, ad libitum energy intake, and 24-h energy expenditure [7]. Keller et al. [6] studied 10 adult males during artificially induced hyperosmolality and hypo-osmolality. Similar to acute fasting, acute hypo-osmolality could result in protein sparing characterized by increased lipolysis, ketogenesis, and lipid oxidation. In contrast, hyperosmolality promoted glycogenolysis and inhibited lipolysis. Moreover, participants’ plasma glucose concentrations increased during hyperosmolality and decreased during hypo-osmolality, in accordance with the recent evidence suggesting a correlation between dehydration and type II diabetes mellitus.

Although current research suggests that AVP and hydration are associated with alterations in one’s nutrient metabolism, these attempts could not take a holistic view of associated metabolic changes. The human body is a complex system functioning with a vast number of metabolic pathways, and these pathways are closely intertwined. Furthermore, the proper function of these metabolic activities does not solely determine one’s wellbeing. Instead, internal physiologic states may interact with one’s living habits and external environmental factors. Therefore, to supplement laboratory-based research on the fundamental mechanism, a holistic approach is required for both breadth and in-depth exploration of the relationship between the hydration state and health in real-world circumstances.

Metabolomics is an evolving approach that enables the assessment of dynamic changes in endogenous metabolites and the in-depth exploration of underlying physiologic and pathologic mechanisms [8]. In recent decades, metabolomic approaches have found applications in the clinical, pharmacologic, and toxicologic realms. In clinical medicine, metabolomics is adopted to discover new diagnostic biomarkers and potential treatment targets for various diseases. Urine metabolomics, in particular, due to its noninvasive and cost-effective nature, has gained increasing attention as a convenient tool for clinical screening, diagnosis, and monitoring of diseases, including cancers [[9], [10], [11]]. Pharmacologic researchers may implement metabolomic analysis to understand the action mechanism and effect of a drug [12]. The impacts of environmental pollutants on one’s health could also be examined and quantified via metabolomic approaches [13].

In the nutritional field, the metabolomics technique enables the investigation of different nutrients or dietary patterns and associated biological changes and health outcomes at the molecular level, thereby promoting the development of public and precision nutrition [14,15]. However, metabolomics remains a relatively new technique in nutrition-related research, and rare research evidence is found on the association between metabolic variations and different hydration states. Therefore, in the current study, we collected urine samples from ad libitum young adults and conducted the current metabolomic analysis to discover variations in the urine metabolome and metabolic pathways that may be associated with one’s hydration state.

## Methods

### Study designs

A 7-d cross-sectional study on fluid intake behavior and the hydration state was implemented from December 9 to 15, 2021, in Baoding, Hebei, China.

### Ethics

The study has been registered with the Chinese Clinical Trial Registry. The registration number is ChiCTR2100045268.

The Peking University Institutional Review Committee has reviewed and approved the study protocol. The ethical approval project identification code is IRB00001052-21013.

### Subjects

Healthy males and females aged 18–25 y from a university in Baoding, Hebei, China, were recruited by convenient and snowball sampling. Smokers, habitual alcohol consumers (>20 g/d), and subjects who had gastrointestinal, oral, and other chronic diseases were excluded from the study. Informed consent was obtained from all subjects.

During the survey, all subjects lived on campus and consumed meals at the university cafeteria explicitly assigned by the research team. Subjects were also prescribed only to perform light physical activity (<3 metabolic equivalents, METS), such as casual walking and stretching, to minimize the impact of physical activity on the hydration state.

### Anthropometric measurements

Height and weight were measured by trained investigators using a height–weight meter (HDM-300). Participants were required to wear light clothing and be barefoot. Height and weight were measured twice. The mean height and weight were then recorded to the nearest 0.1 cm and 0.1 kg.

BMI was calculated with the following equation: BMI = weight(kg)/height^2^(m). The classification criteria were based on the guidelines established by the Working Group on Obesity in China as follows: *1*) normal (18.5 kg/m^2^ ≤ BMI < 24.0 kg/m^2^), *2*) underweight (<18.5 kg/m^2^), *3*) overweight (24.0 kg/m^2^ ≤ BMI < 28.0 kg/m^2^), and *4*) obese (≥28.0 kg/m^2^) [16].

### Temperature and humidity

Temperature hygrometers (No. 8813, Deli) were placed at the sports ground, classroom, and dormitory. The indoor and outdoor measurements were recorded daily to the nearest 0.1°C and 1% by trained investigators at 10:00, 14:00, and 20:00.

### Collection of 24-h urine

Participants’ 24-h urine samples were collected for 3 consecutive days from December 9 to 11, 2021, using custom urine collectors (graduated urine bags). To collect 24-h urine samples, participants’ first voided morning urine was discarded. Subsequent urine produced during the day and the first voided morning urine of the next day were included [17]. All collected urine samples were immediately kept refrigerated at 4°C. The 24-h urine samples from day 2 were used for the liquid chromatography-MS (LC-MS) analysis.

### Classification of the hydration state

Urine osmolality was measured using an osmometer, strictly following the instruction manual.

In the current study, subjects were divided into 3 groups based on the 3-d means of 24-h urine osmolality: *1*) urine osmolality ≤ 500 mOsm/kg was classified as optimal hydration (OH), *2*) 500–800 mOsm/kg was classified as middle hydration (MH), and *3*) >800 mOsm/kg was classified as hypohydration (HH) [18,19].

### Urine metabolomic analysis

#### Chemicals and reagents.

LC-MS-grade acetonitrile (ACN) and HPLC-grade methanol (MeOH) were purchased from Merck. Distilled water was used throughout all procedures.

#### Urine samples preparation.

In a centrifuge tube, 50 μL of urine sample was mixed with 450 μL of precipitant to precipitate urine protein. The mixture was centrifuged (12,000 g; 10 min; 4°C). Then, 100 μL of the supernatant was transferred for further LC-MS/MS^2^ analysis.

#### LC-MS conditions.

Sample extracts were analyzed using an ultra–high-performance liquid chromatography system (Waters ACQUITY BEH C18 1.7 μm, 2.1 × 50 mm, www.waters.com; MS, DIONEX Ultimate 3000, www.thermofisher.com). The condition of the liquid phase is shown in Table 1.

**TABLE 1:** Gradient conditions.

Time (min)	A (%)	D (%)
0	95	5
1.00	95	5
5.00	40	60
8.00	0	100
11.00	0	100
14.00	40	60
15.00	95	5
18.00	95	5

A: water (includes 2 mmol/L ammonium formate and 0.1% formic acid); D: acetonitrile; gradient elution; flow rate 0.25 mL/min.

#### Visualization of raw chromatogram.

**FIGURE 1:**
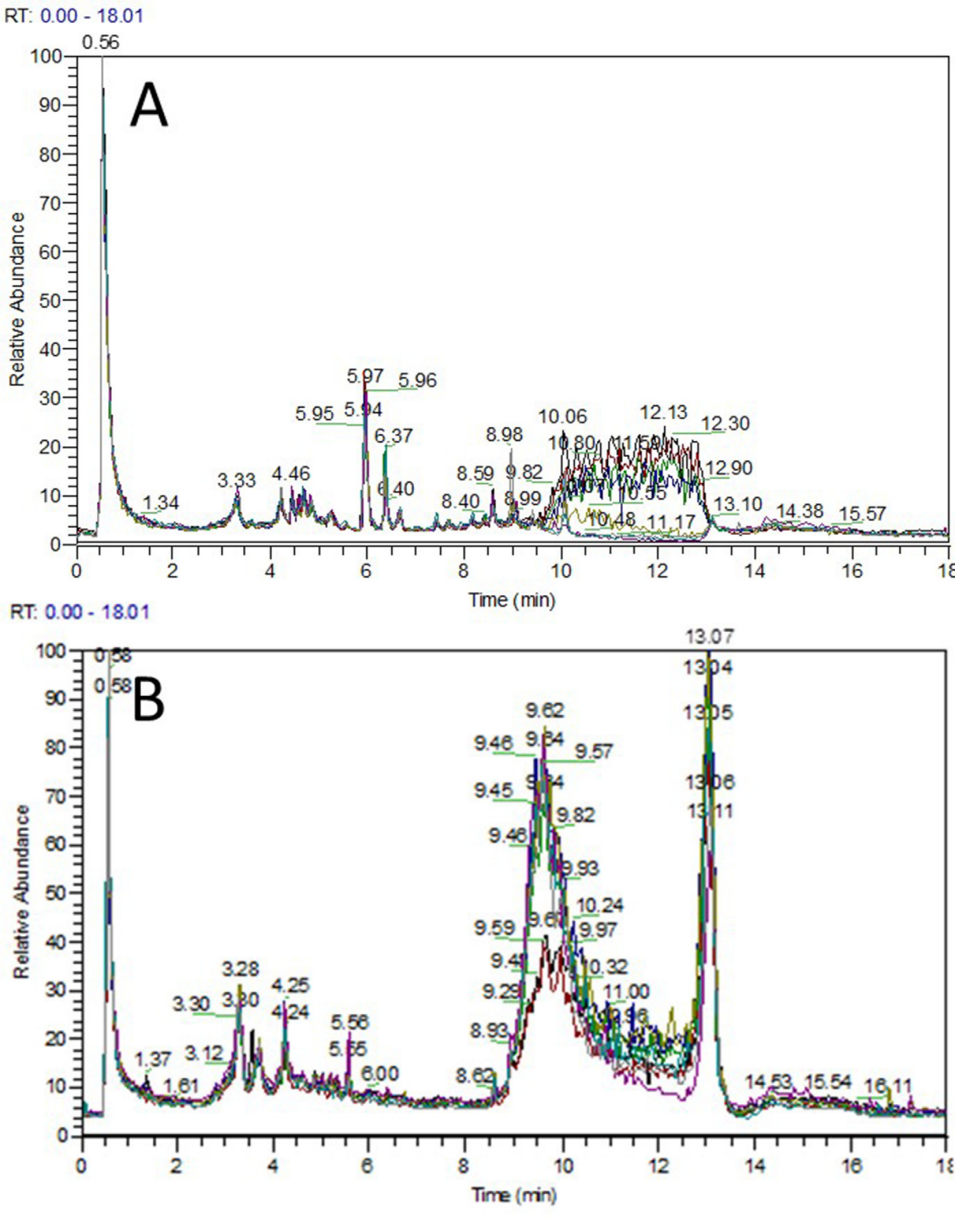
(A) Cations total ion chromatogram of quality control samples. (B) Anions total ion chromatogram of quality control samples.

**FIGURE 2:**
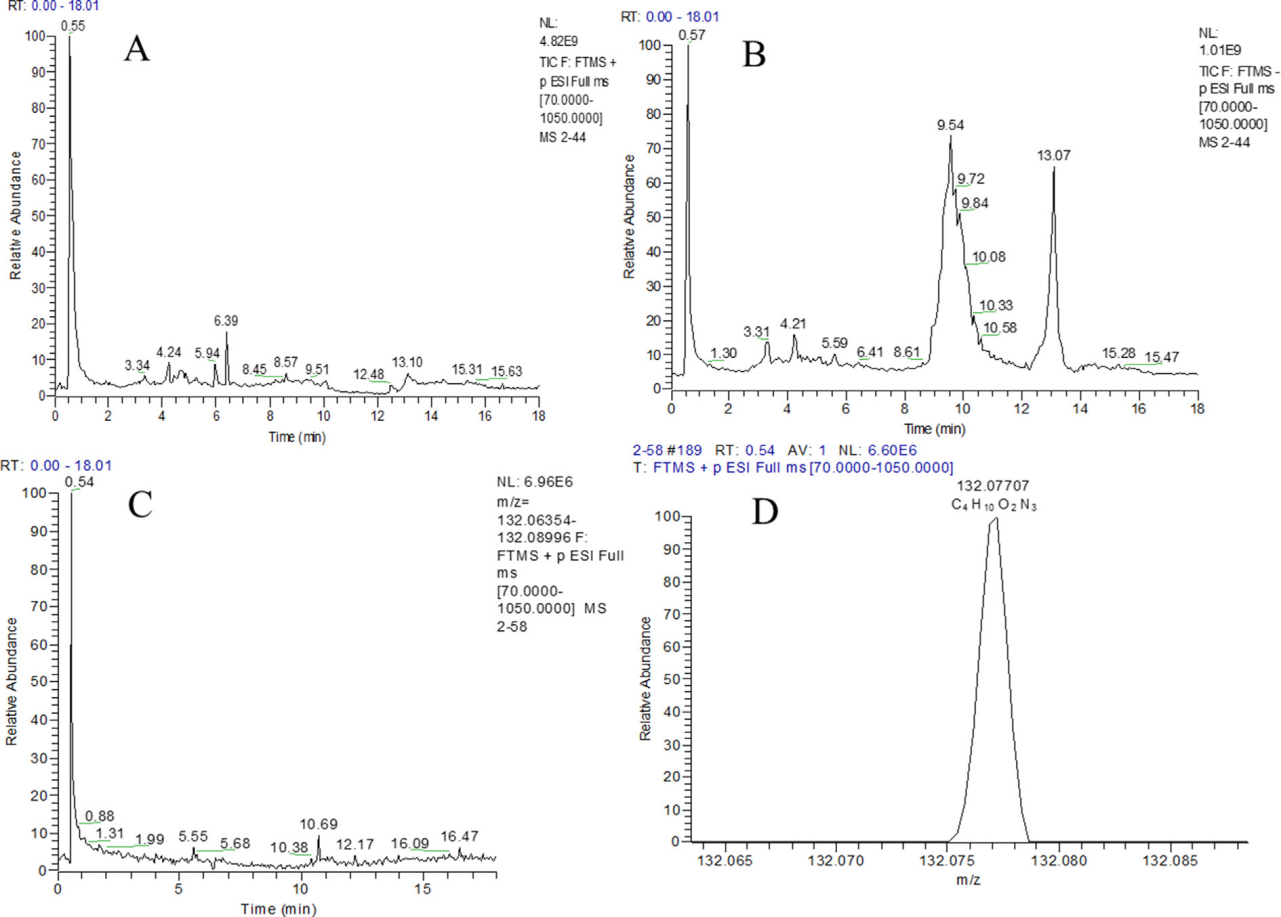
(A) Full scan cations total ion chromatogram of urine samples. (B) Full scan anions total ion chromatogram of urine samples. (C) Chromatogram of creatine. (D) Accurate mass and molecular formula of creatine.

Figure 1 shows the total ion current chromatogram of quality control samples. The retention time reproducibility of the instrument indicated that the instrument was stable, thus improving the reliability of instrumental analysis and data. The full scan mode was then used to analyze the sample homogenate, and the total ion current chromatogram of urine samples is shown in Figure 2A, B.

### Statistical analysis

#### Baseline characteristics.

SPSS 26.0 was used for statistical analysis of baseline characteristics and the hydration state. Continuous variables were analyzed using the *t*-test and one-way analysis of variance (ANOVA). Results are presented as mean ± SD. Categorical variables were compared using the Fisher’s exact test. Results are presented as *n* (%). All statistical tests were 2-sided. A significance level < 0.05 represents a statistically significant difference.

#### Data processing.

LC-MS data were preprocessed using the self-built database of TraceFinder software (www.thermofisher.com). Sample parameters were acquired, including variables (retention time_mass charge ratio), molecular weight, observed value, and peak intensity. The data matrix was then imported to MetaboAnalyst 5.0 (www.metaboanalyst.ca) and underwent normalization by median, log transformation, and Pareto scaling using built-in features of MetaboAnalyst 5.0 before subsequent analysis.

#### Correlational analysis

The hierarchical cluster analysis was conducted to visualize the correlation between different hydration states and urine metabolome.

#### Unsupervised and supervised discriminant analysis

The unsupervised principal component analysis (PCA) was implemented to estimate group differences and individual variations. Scree and score plots were generated for all pairwise and 3-way comparisons. We also conducted 2 supervised discriminant analyses, partial least squares discriminant analysis (PLS-DA), and orthogonal projections to latent structures discriminant analysis (OPLS-DA), to further enhance the ability of multidimensional data clustering and modeling. Similar to PLS-DA, OPLS-DA is a supervised algorithm that eliminates and isolates the variations of independent variables irrelevant to the variations of dependent variables. Thereby, the intergroup differences are maximized. The model’s ability to explain the variation of the dependent variable is enhanced as well. Furthermore, PLS-DA provides an essential and commonly used reference parameter while screening for differential metabolites, the variable importance in projection (VIP).

#### Metabolites and pathways analysis.

The VIP values for detected metabolites were generated from OPLS-DA. Metabolites with VIP > 1 were selected for subsequent *t*-test and fold change (FC) analysis to compute the false discovery rate (FDR), the adjusted *P* value for multiple comparisons that lowers the probability of false positives. A volcano plot was generated with log2(FC) as the *X* axis and −log10(FDR) as the *Y* axis. Metabolites that had both FC > 2 (or FC < 0.5) and FDR < 0.05 were uploaded to the Human Metabolome Database (HMDB), enabling the screening for differential endogenous metabolites.

We uploaded the 85 differential endogenous metabolites identified by previous analysis to the Kyoto Encyclopedia of Genes and Genomes (KEGG) databases to perform the pathway enrichment analysis. A bubble plot was generated with the pathway impact as the *X* axis and the *P* value as the *Y* axis. The pathway impact was calculated with pathway topology analysis by adding the importance measures of each matched metabolite and then dividing by the sum of the importance measures of all metabolites for each pathway. In this case, the relative betweenness centrality, which is defined as the number of shortest paths going through the node, was adopted as the importance measure.

**FIGURE 3:**
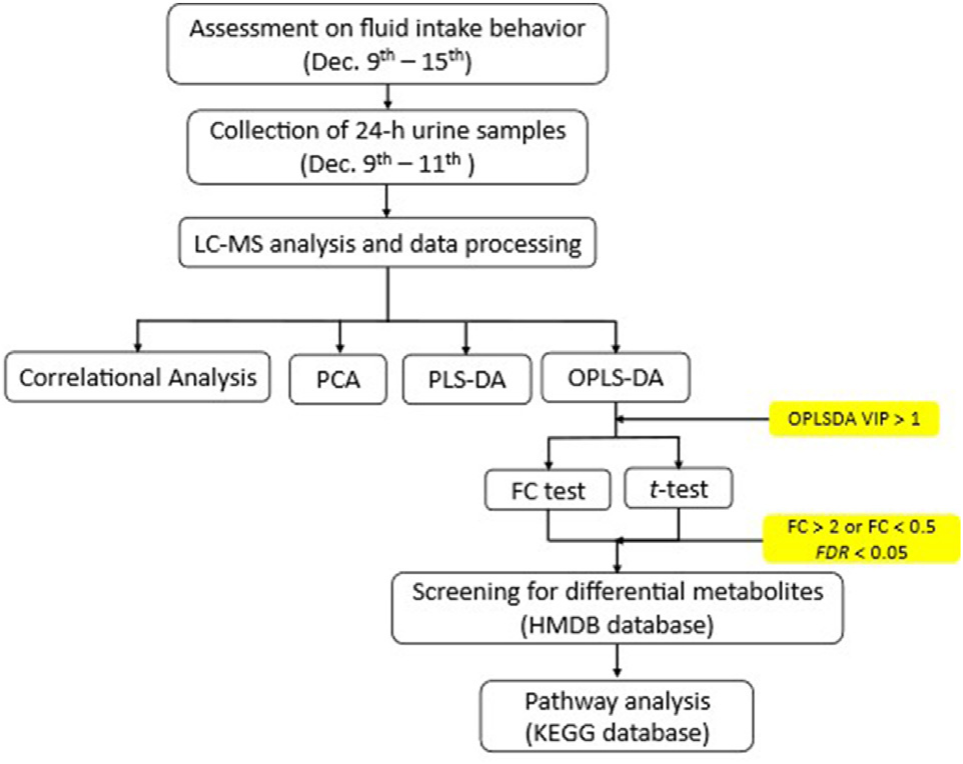
A flow chart of the study protocol.

A flow chart of the study protocol is shown in Figure 3.

## Results

### Temperature and humidity

From December 9 to 11, 2021, the average indoor and outdoor temperatures were 22.4°C (72.3°F) and 4.9°C (40.8°F). The average indoor and outdoor humidity was 44% and 79%.

### Characteristics of subjects

In total, 79 subjects participated in the fluid intake behavior assessment from December 9 to 15, 2021, among which 67 24-h urine samples were collected from December 9 to 11, 2021, for further analysis. Among the 67 participants in the current metabolomic analysis, 43 were males and 24 were females. The average age was 19.9, and the average BMI was 23.8 kg/m^2^.

As shown in Table 2, the mean urine osmolality for days 1, 2, and 3 were 591.33 ± 240.22, 616.45 ± 219.67, and 624.01 ± 216.912 mOsm/kg (*P* > 0.05), respectively. The mean 3-d average urine osmolality was 610.60 ± 198.21 mOsm/kg. No significant difference in mean urine osmolality was found between each day and the 3-d average.

**TABLE 2:** The urine osmolality of subjects (mOsm/kg).

		Day 1	Day 2	Day 3	3-d average
Overall		591.33	616.45	624.01	610.60
Sex	Male (*n* = 43)	621.30	624.37	641.12	628.93
	Female (*n* = 24)	537.63	602.25	593.38	577.75
BMI (kg/m^2^)	Normal (*n* = 34)	582.55	648.70	604.36	611.90
	Underweight (*n* = 8)	477.29	588.43	622.14	562.62
	Overweight (*n* = 14)	610.00	599.50	638.29	615.93
	Obese (*n* = 13)	654.92	567.69	659.54	627.38

Abbreviations: BMI, body mass index.

Subjects were divided into OH (*n* = 21), MH (*n* = 33), and HH (*n* = 13) groups based on their 3-d average urine osmolality. Table 3 summarizes the characteristics of subjects in different groups. Neither sex, age, nor BMI was significantly associated with one’s hydration state.

**TABLE 3:** The characteristics of subjects.

	Overall	OH (*n* = 21)	MH (*n* = 33)	HH (*n* = 13)	*P*
Height (cm)	169.54 ± 7.68	168.01 ± 7.73	171.85 ± 6.79	172.35 ± 7.96	0.125
Weight (kg)	67.50 ± 16.07	63.09 ± 17.28	73.19 ± 15.79	71.94 ± 13.50	0.072
BMI (kg/m^2^)	23.36 ± 4.77	22.14 ± 4.83	24.70 ± 4.76	24.31 ± 5.10	0.163
Systolic pressure	114.22 ± 10.76	111.81 ± 12.07	117.12 ± 8.75	117.08 ± 10.22	0.149
Diastolic pressure	75.62 ± 6.72	74.52 ± 7.28	77.18 ± 6.91	74.23 ± 4.36	0.238

Abbreviations: BMI, body mass index; HH, hypohydration; MH, middle hydration; OH, optimal hydration.

### Correlational analysis

**FIGURE 4:**
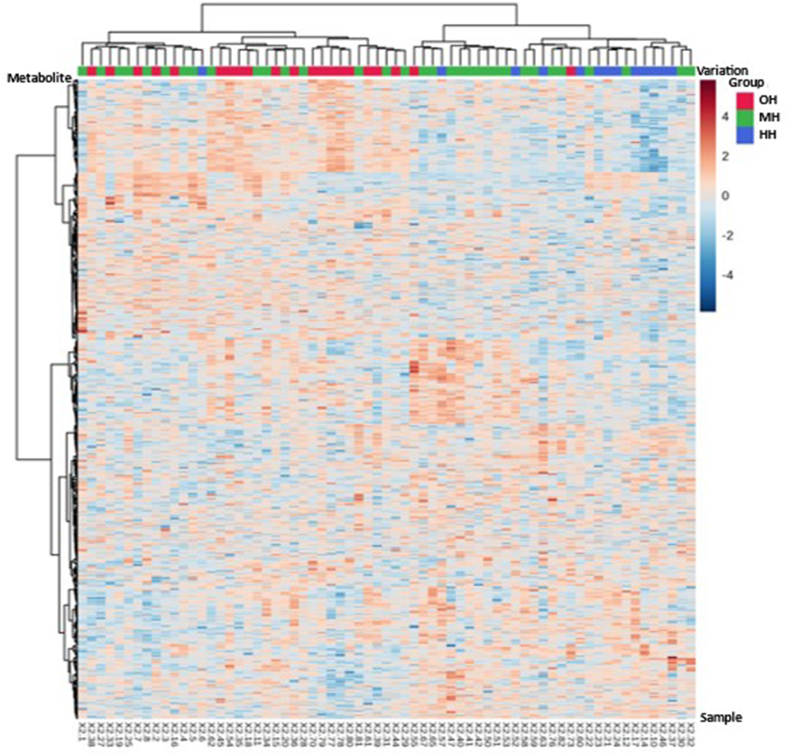
The clustered correlational heatmap of samples with urine samples as the *X* axis and metabolites as the *Y* axis. The grouping information of each sample was shown by the colors of the bands at the top of the diagram. A deeper color block in the diagram reflects a more significant amount of change in the concentration of a metabolite. A warmer tone stands for a positive correlation, and a cooler tone stands for an adverse correlation.

We identified 1055 metabolites based on the untargeted metabolomic approach. A clustered correlational heatmap of metabolites and samples, visualizing the clustering trend of samples with various hydration states, is shown in Figure 4. Samples from the OH, MH, and HH groups were colored in red, green, and blue, respectively. The dendrogram of sample clustering showed a noticeable separation trend between the OH and HH groups. Samples of the OH group were mainly clustered at the left branch. Samples of the HH group were mostly clustered at the right branch of the dendrogram. Samples of the MH group were evenly distributed in both major branches. Together, we found that the urinary concentrations of metabolites in the upper right quadrant decreased as the hydration state of samples became less optimal, in contrast to metabolites in the lower left quadrant. Meanwhile, the urinary concentrations of metabolites in the upper left quadrant increased as the hydration state improved, in contrast to metabolites in the lower right quadrant.

### PCA analysis

**FIGURE 5:**
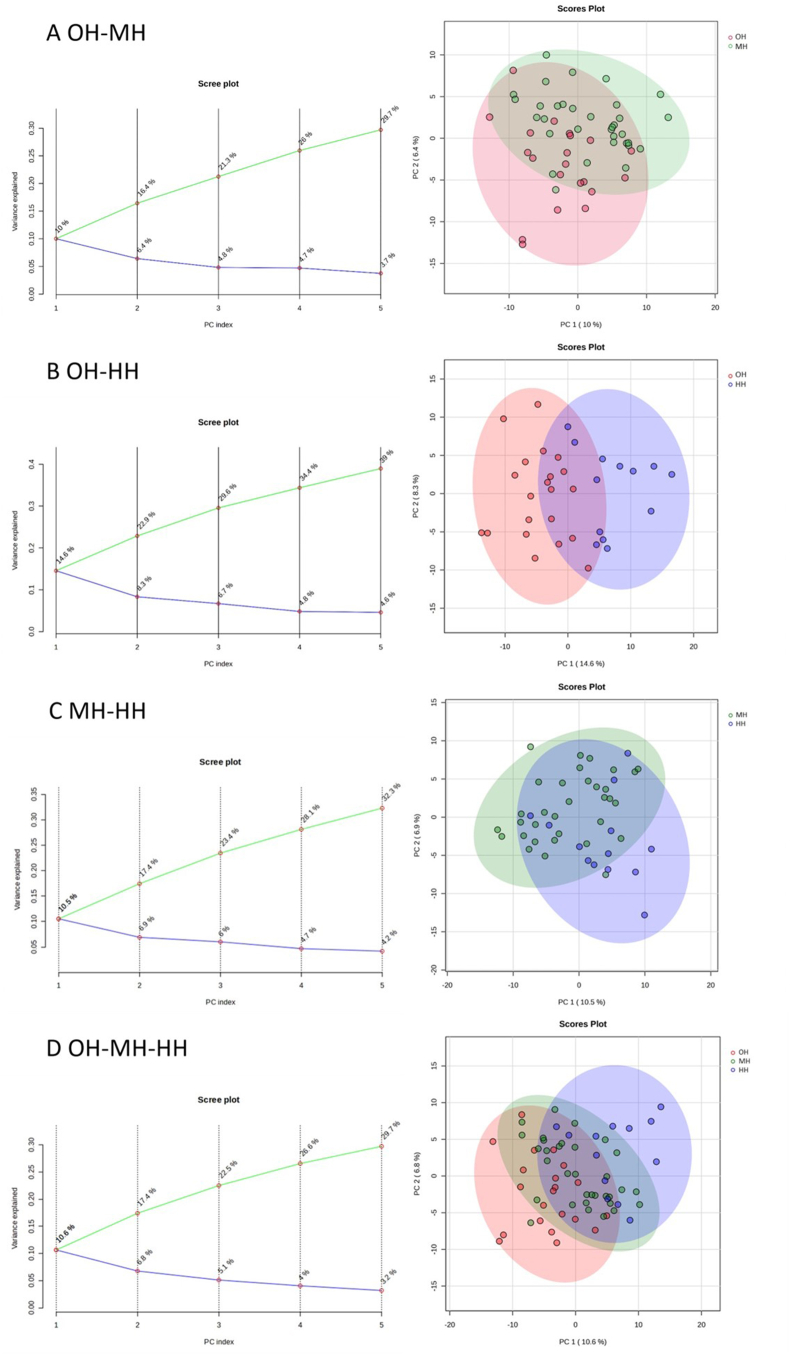
(A) PCA plots of the optimal hydration group and middle hydration group. (B) PCA plots of the optimal hydration group and hypohydration group. (C) PCA plots of the middle hydration group and hypohydration group. (D) PCA plots of all 3 groups. PCA, principal component analysis.

Figure 5A–D shows the scree and score plots generated for 3 pairwise comparisons (OH–MH, OH–HH, and MH–HH) and a 3-way comparison (OH–MH–HH). The OH and HH groups showed the best statistically separated trend among all 3 pairwise comparisons. However, the statistical separations were not ideal for all comparison groups.

### PLS-DA analysis

**FIGURE 6:**
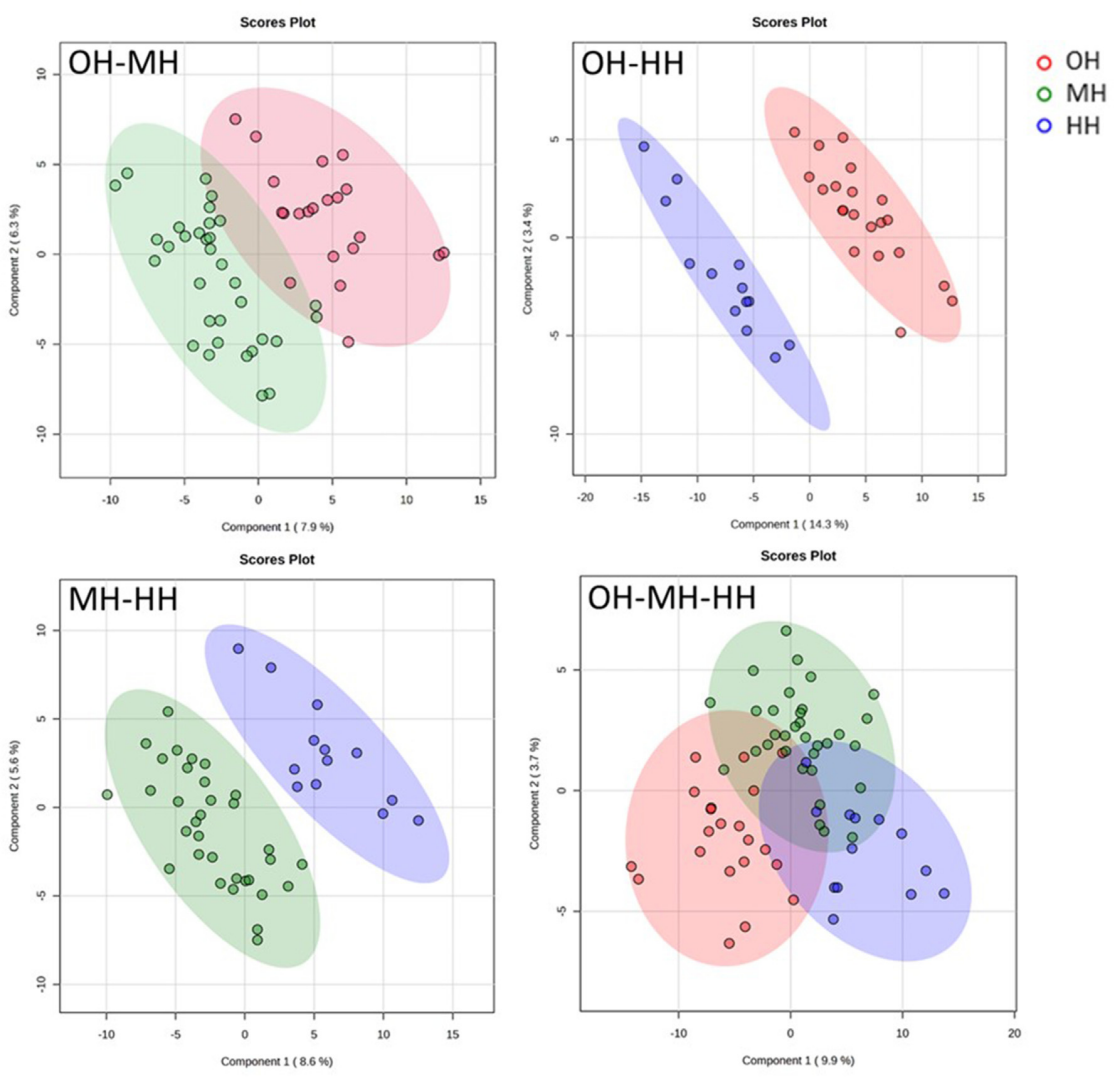
PLS-DA plots of subjects with different hydration states. PLS-DA, partial least squares discriminant analysis.

The score plots of all comparison groups (OH–MH, OH–HH, MH–OH, and OH–MH–HH) are presented in Figure 6. All 4 comparison groups exhibited better statistical separation trends than PCA analysis, especially for the 3-way comparison OH–MH–HH.

The parameters reflecting model quality for each comparison group were also computed using the permutation test, as listed in Table 4. *R*^*2*^ measures the explained variation and the goodness of fit. *Q*^*2*^ measures the predicted variation and the goodness of prediction. As *R*^*2*^ approaches 1, the model fits the data better. A *Q*^*2*^ > 0.5 usually means the model has good predictive ability. In the study, only the OH–HH model had a *Q*^*2*^ = 0.55 and a *R*^*2*^ = 0.74, which means the model could fit and predict the data well. However, the predictive ability and goodness of fit were less desirable for the rest of the generated models. The rest of the models were less likely to correctly distinguish a group from the others.

**TABLE 4:** PLS-DA model parameters.

Measures	OH–MH	OH–HH	MH–HH	OH–MH–HH
Accuracy	0.76	0.91	0.73	0.59
*R*^*2*^	0.56	0.74	0.57	0.47
*Q*^*2*^	0.22	0.55	0.14	0.30

Abbreviations: HH, hypohydration; MH, middle hydration; OH, optimal hydration; PLS-DA, partial least squares discriminant analysis.

### OPLS-DA analysis

**FIGURE 7:**
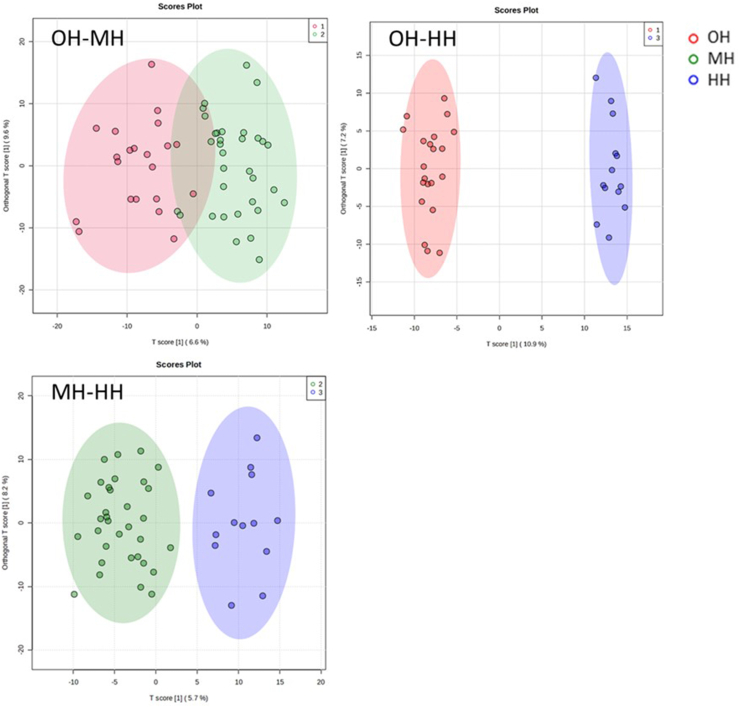
OPLS-DA plots of subjects with different hydration states. OPLS-DA, orthogonal projection to latent structures discriminant analysis.

A score plot was generated for each pairwise comparison (OH–MH, OH–HH, and MH–HH), as shown in Figure 7. Model quality parameters were also computed using the permutation analysis and listed in Table 5.

**TABLE 5:** OPLS-DA model parameters.

Measures	OH–MH	OH–HH	MH–HH
*R*^*2*^*Y*	0.74	0.99	0.85
*P*	1.00	<0.01	0.84
*Q*^*2*^	0.11	0.51	0.125
*P*	0.03	<0.01	0.04

Abbreviations: HH, hypohydration; MH, middle hydration; OH, optimal hydration; OPLS-DA, orthogonal projection to latent structures discriminant analysis; PCA, principal component analysis.

The OPLS-DA plot showed significant improvements in statistical separation for all comparison pairs. However, the predictive model of the OH–HH group still had the best predictive ability and goodness of fit (*R*^*2*^*Y* = 0.99, *Q*^*2*^ = 0.51, *P* < 0.01) among all 3 pairs, suggesting that subjects from the OH and HH groups might have the most distinct metabolic profiles. Given the current data, the statistical separation and discrimination of the MH group from the OH and HH groups were less optimal. Therefore, the following sections will explore the differential metabolites and metabolic pathways between the OH and HH groups.

### Screening of differential metabolites

**FIGURE 8:**
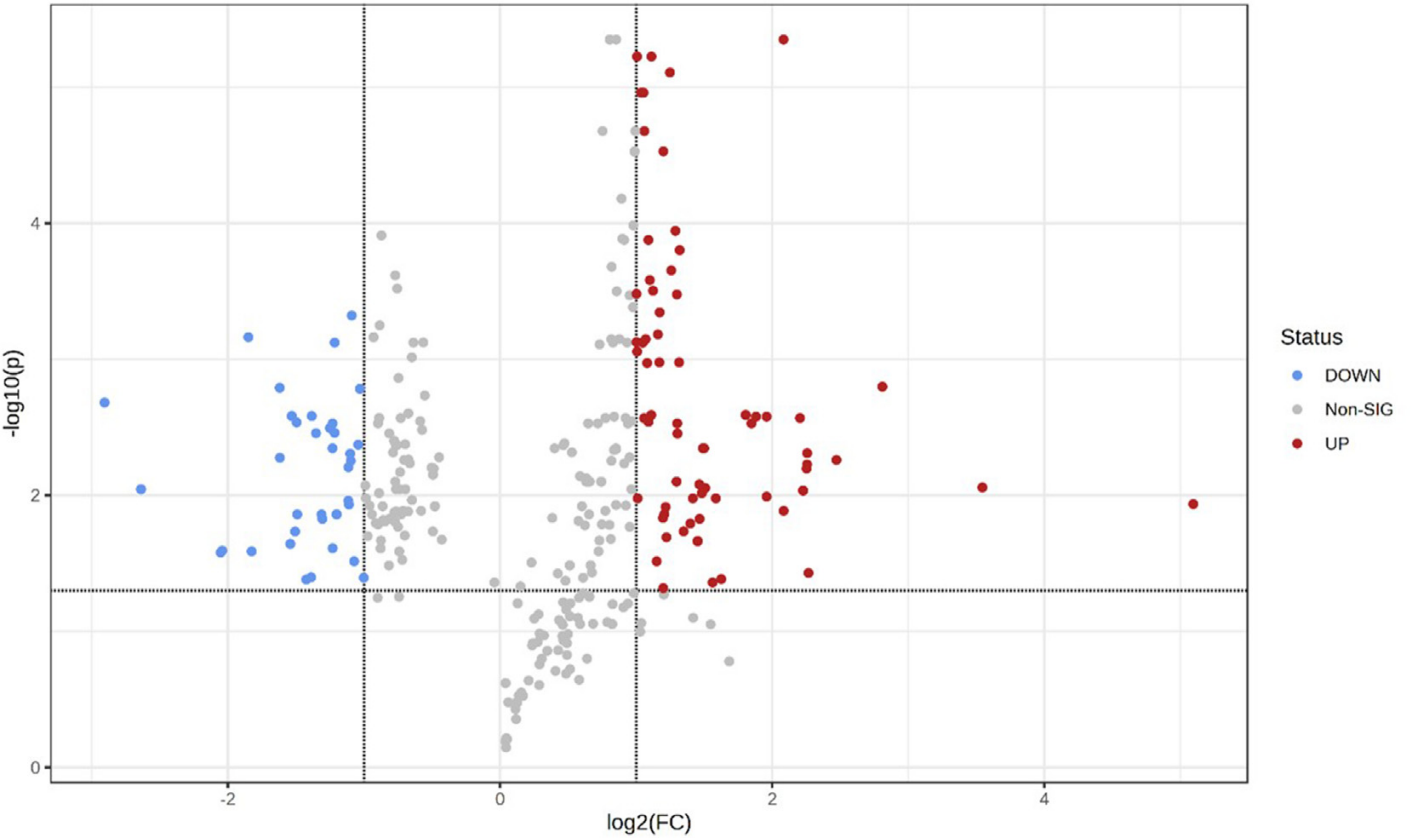
The volcano plot of differential metabolites between the OH and HH groups with fold change as the *X* axis and FDR as the *Y* axis. Blue dots represent metabolites that were significantly downregulated in the OH group than in the HH groups, and red dots represent metabolites that were significantly upregulated in the OH group than in the HH group. FDR, false discovery rate; HH, hypohydration; OH, optimal hydration.

We found that 338 of the 1055 detected metabolites had the OH–HH OPLS-DA VIP > 1. The volcano plot is shown in Figure 8. According to the results of the *t*-test and FC test, 115 metabolites had both FC > 2 (or FC < 0.5) and *FDR* < 0.05. The intensity peaks of 40 metabolites were significantly downregulated, and 75 were significantly upregulated in the OH group. Among the 115 metabolites, 85 were recorded by HMDB. The results are listed in Table 6.

**TABLE 6:** Differential endogenous metabolites between the OH and HH groups.

Significantly down				Significantly up			
HMDB ID	Compound	FC (OH/HH)	FDR	HMDB ID	Compound	FC (OH/HH)	FDR
0005039	Sildenafil	0.13	0.002	0029206	lysoPC(28:0)	34.17	0.012
0000543	Benzenebutanoic acid	0.16	0.009	0008362	PC(20:3(5Z,8Z,11Z)/14:1(9Z))	11.67	0.009
0032891	1-(4-Methoxyphenyl)-2-propanone	0.16	0.009	0007887	PC(14:0/22:1(13Z))	7.01	0.002
0000574	L-Cysteine	0.28	0.001	0010168	SM(d18:0/16:0)	5.55	0.005
0011504	LysoPE(16:1(9Z)/0:0)	0.34	0.023	0008014	PC(16:1(9Z)/20:3(8Z,11Z,14Z))	4.81	0.037
0011474	LysoPE(0:0/16:1(9Z))	0.34	0.023	0007899	PC(14:1(9Z)/14:0)	4.78	0.006
0001220	Prostaglandin E2	0.35	0.003	0013333	3-Hydroxy-9-hexadecenoylcarnitine	4.77	0.006
0002776	13,14-Dihydro-15-keto-PGE2	0.35	0.003	0004950	Ceramide (d18:1/18:0)	4.68	0.009
0006236	Phenylacetaldehyde	0.35	0.018	0011762	Cer(d18:0/18:1(11Z))	4.68	0.009
0035078	Geranial	0.38	0.003	0008049	PC(18:0/20:4(8Z,11Z,14Z,17Z))	4.61	0.003
0011477	LysoPE(0:0/18:2(9Z,12Z))	0.40	0.014	0030707	4,5-Di-O-caffeoylquinic acid	4.24	0.013
0033897	Ginkgolic acid	0.42	0.003	0013463	SM(d18:0/16:1(9Z)(OH))	4.24	<0.001
0003252	Thromboxane B2	0.43	0.003	0015240	Trilostane	3.89	0.003
0000153	Estriol	0.43	0.001	0013462	SM(d18:0/14:1(9Z)(OH))	3.69	0.003
0001425	Estrone sulfate	0.43	0.014	0010570	PG(16:0/16:0)	3.60	0.003
0003306	Phloretin	0.44	0.014	0000192	L-Cystine	3.50	0.003
0036156	Vomitoxin	0.46	0.006	0012252	Linoleoyl ethanolamide	2.95	0.044
0000086	Glycerophosphocholine	0.46	0.012	0010169	SM(d18:1/16:0)	2.84	0.009
0005083	8-Isoprostaglandin F2a	0.47	0.006	0000268	Tetrahydrocorticosterone	2.83	0.005
000159	L-Phenylalanine	0.47	<0.001	0005972	Tetrahydrodeoxycortisol	2.81	0.005
0029667	2,3,6-Trimethylphenol	0.48	0.031	0011567	MG(18:1(9Z)/0:0/0:0)	2.79	0.010
0003464	4-Guanidinobutanoic acid	0.49	0.004	0007920	PC(14:1(9Z)/22:1(13Z))	2.76	0.015
				0001859	Acetaminophen	2.76	0.008
				0010658	PG(18:2(9Z,12Z)/22:5(7Z,10Z,13Z,16Z,19Z))	2.74	0.022
				0010657	PG(18:2(9Z,12Z)/22:5(4Z,7Z,10Z,13Z,16Z))	2.74	0.022
				0010644	PG(18:1(9Z)/22:6(4Z,7Z,10Z,13Z,16Z,19Z))	2.74	0.022
				0004951	Ceramide (d18:1/20:0)	2.67	0.011
				0000101	Deoxyadenosine	2.64	0.016
				0010680	PG(18:3(9Z,12Z,15Z)/18:2(9Z,12Z))	2.55	0.018
				0013410	PC(o-16:1(9Z)/14:1(9Z))	2.50	<0.001
				0007873	PC(14:0/18:1(9Z))	2.46	<0.001
				0000733	Hyodeoxycholic acid	2.46	<0.001
				0002005	Methionine sulfoxide	2.39	<0.001
				0061007	N-Desmethyltramadol	2.38	<0.001
				0001565	Phosphorylcholine	2.33	0.020
				0013464	SM(d18:0/16:1(9Z))	2.29	0.015
				0004947	Ceramide (d18:1/12:0)	2.24	0.001
				0010384	LysoPC(18:0)	2.22	0.031
				0013411	PC(o-16:1(9Z)/16:1(9Z))	2.18	<0.001
				0007911	PC(14:1(9Z)/20:0)	2.16	0.003
				0032291	Geranyl diphosphate	2.15	<0.001
				0007864	PA(18:1(9Z)/18:1(11Z))	2.13	0.003
				0007862	PA(18:1(11Z)/18:1(11Z))	2.13	0.003
				0035396	T2 Triol	2.13	0.000
				0002005	Methionine sulfoxide	2.12	0.001
				0002434	Hydroquinone	2.08	0.003
				0034159	Acetyl tributyl citrate	2.08	<0.001
				0000830	Neuraminic acid	2.07	0.001
				0000085	Deoxyguanosine	2.07	0.001
				0000050	Adenosine	2.07	0.001
				0038848	Rhoifolin	2.02	0.011
				0030290	Cuscohygrine	2.01	<0.001
				0014334	Bortezomib	2.00	0.001

Abbreviations: FC, fold change; FDR, false discovery rate; HH, hypohydration; HMDB, Human Metabolome Database; OH, optimal hydration.

### Pathway analysis

**FIGURE 9:**
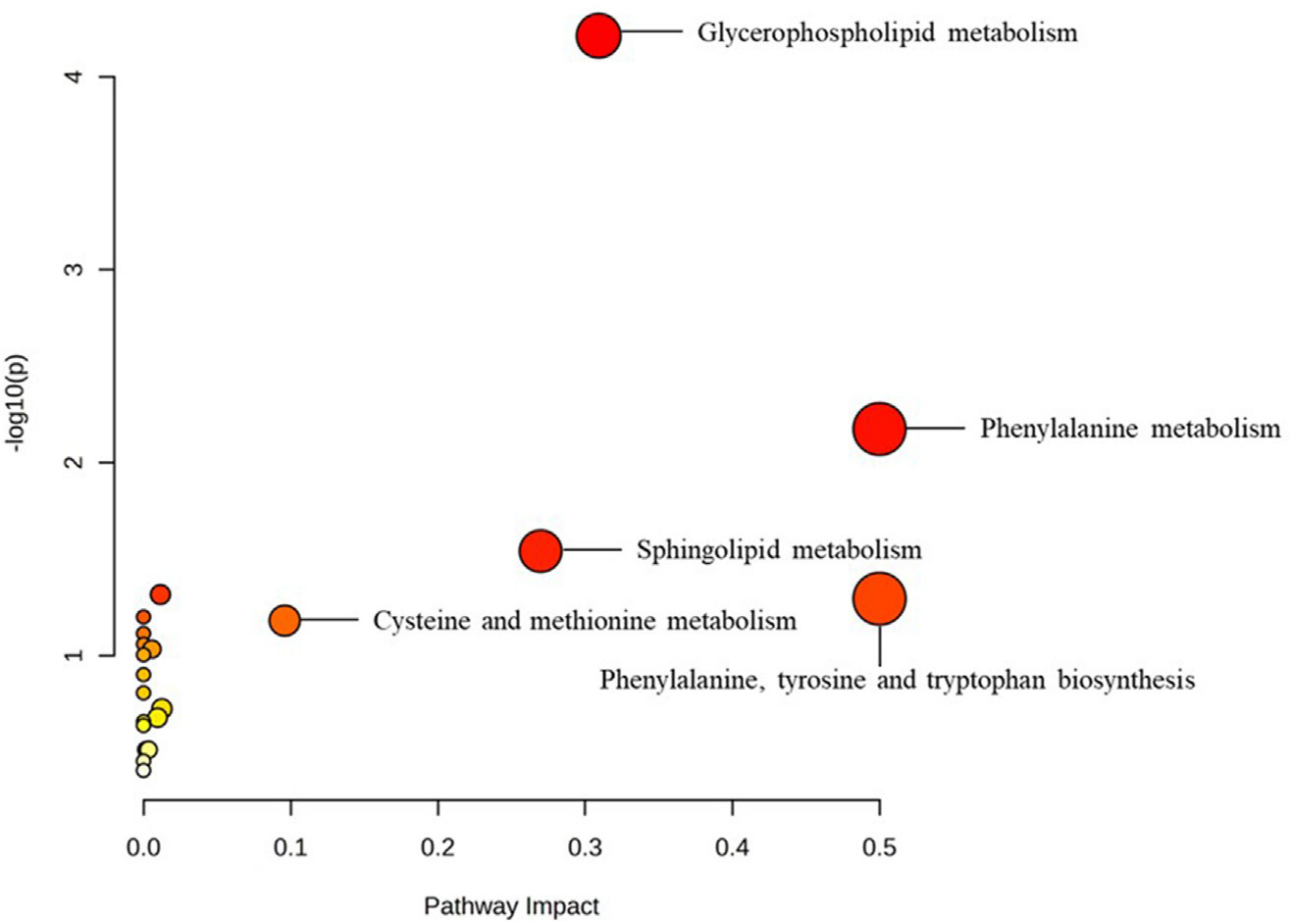
Pathway impact (*X* axis) and statistical significance (*Y* axis) of the metabolic pathways identified through pathway analysis of 85 differential endogenous metabolites. A deeper color represents a lower chance of false positives, and a larger circle represents a more significant pathway impact.

We identified 21 pathways as candidates for further investigation. A list of screened pathways and corresponding statistical parameters is shown in Table 7. A bubble plot with the pathway impact and statistical significance of each pathway as the *X* axis and *Y* axis is presented in Figure 9. As a result of the pathway impact analysis, the phenylalanine metabolic pathway and the phenylalanine, tyrosine, and tryptophan biosynthesis pathway had the most significant pathway impact, followed by the glycerophospholipid metabolic pathway, the sphingolipid metabolic pathway, and the cysteine and methionine metabolic pathway. The KEGG pathway maps of the phenylalanine metabolism, the phenylalanine, tyrosine, and tryptophan biosynthesis, and the glycerophospholipid metabolism are shown in Supplemental Tables 1–3, respectively. Affected pathway nodes are labeled in red.

**TABLE 7:** Potential metabolic pathways identified by pathway analysis.

	Total number	Expected hits	Actual hits	*P*	FDR	Impact
Phenylalanine metabolism	10	0.13	2	0.007	0.281	0.50
Phenylalanine, tyrosine, and tryptophan biosynthesis	4	0.05	1	0.051	0.755	0.50
Glycerophospholipid metabolism	36	0.46	5	0.000	0.005	0.31
Sphingolipid metabolism	21	0.27	2	0.029	0.755	0.27
Cysteine and methionine metabolism	33	0.43	2	0.066	0.755	0.10
Glycerolipid metabolism	16	0.21	1	0.188	1.000	0.01
Purine metabolism	65	0.84	3	0.048	0.755	0.01
Terpenoid backbone biosynthesis	18	0.23	1	0.209	1.000	0.01
Steroid hormone biosynthesis	85	1.10	3	0.092	0.755	0.01
Glutathione metabolism	28	0.36	1	0.307	1.000	0.00
Phosphatidylinositol signaling system	28	0.36	1	0.307	1.000	0.00
Linoleic acid metabolism	5	0.06	1	0.063	0.755	0.00
Arachidonic acid metabolism	36	0.46	2	0.077	0.755	0.00
Thiamine metabolism	7	0.09	1	0.087	0.755	0.00
Taurine and hypotaurine metabolism	8	0.10	1	0.099	0.755	0.00
Aminoacyl-tRNA biosynthesis	48	0.62	2	0.125	0.878	0.00
α-Linolenic acid metabolism	13	0.17	1	0.156	1.000	0.00
Pantothenate and CoA biosynthesis	19	0.25	1	0.220	1.000	0.00
Ether lipid metabolism	20	0.26	1	0.230	1.000	0.00
Glycine, serine, and threonine metabolism	33	0.43	1	0.352	1.000	0.00
Arginine and proline metabolism	38	0.49	1	0.393	1.000	0.00

Abbreviation: FDR, false discovery rate.

## Discussion

The present study explored variations in urine metabolome among subjects with different hydration states classified by one’s 24-h urine osmolality via an untargeted LC-MS approach. We did not find a significant impact of the dates of sample collection and subjects’ characteristics on subjects’ hydration state. Although previous studies reported seasonal variations in the hydration state and fluid intake behavior, our results suggested that one’s hydration state and fluid intake behavior were unlikely to vary significantly over 3 d due to the relatively consistent fluid intake habits.

Regarding the association between the hydration state and urine metabolome, results from previous analyses, including the correlational analysis, PCA, PLS-DA, and OPLS-DA, all suggested a clear statistical separation trend between samples from the OH and HH groups. However, samples from the MH group were less distinguishable from the former groups. In line with the graphical visualization, the parameters of model qualities computed from both unsupervised and supervised analyses of the pairwise comparison OH–HH were of the best goodness of fit and predictive ability among all comparison groups.

Therefore, we screened for differential metabolites between the OH and HH groups. One hundred fifteen metabolites that had OPLS-DA VIP > 1 were selected out of 338 metabolites according to the FC and FDR. Among the 115 identified metabolites, we found a significant association between the hydration state and the urinary concentration of metabolites associated with phospholipids (PLs), lysophospholipids (LPLs), and amino acids metabolism. Although the hydration state was traditionally linked to kidney conditions, our finding suggested that there might be underlying biological changes in multiple neuropsychologic and metabolic conditions associated with the hydration state [1].

The urinary concentrations of lysophosphatidylcholines (lysoPC) (28:0) were 34 times higher in the OH group than in the HH group. Other members of PLs such as PC(20:3(5Z,8Z,11Z)/14:1(9Z)) and PC(14:0/22:1(13Z)) also had leading FC. Results from the pathway analysis suggested that the glycerophospholipid metabolic pathway and the sphingolipid metabolic pathway were also significantly impacted by variations in the hydration state, with leading impact factors equal to 0.31 and 0.27. PC is a class of PLs with a choline headgroup. LysoPC are hydrolyzed PC that also dominates the PL files in blood. PLs are involved in diverse physiologic functions, including energy metabolism, apoptosis, and signal transduction. Due to their noninvasive and convenient nature, urinary PLs are attracting increasing attention as predictive and complementary clinical biomarkers for various cancers and other diseases. PC, in particular, is the primary dietary source of choline, therefore taking up an irreplaceable role in choline metabolism within the body. Alteration in choline metabolism could be associated with the onset and progression of nonalcoholic fatty liver diseases [20]. Research evidence also suggests that PC concentrations in the brain are associated with cognitive function. Although PC concentrations in the brain may slowly decrease while aging, researchers found a reduction in PC concentrations in the brains of patients with Alzheimer’s disease [[21], [22], [23]].

In addition, our finding suggested that urinary sphingomyelin (SM) and ceramide were significantly upregulated in the OH group. SM and ceramide are versatile molecules found in cell membranes, particularly the membranes of peripheral and central nerve cells, serving a variety of crucial physiologic functions, including regulating cell division and differentiation. Similar to PC, dysregulated SM metabolism is associated with neurologic, cardiovascular, and respiratory conditions [24]. Previous studies have pointed out the potential correlation between one’s hydration states and cognitive function, yet the mechanism of action has not been thoroughly studied yet [25,26]. Given the evidence that PC concentrations have a long-term impact on cognitive function and that urinary PC concentrations could be affected by the short-term hydration states, whether PC could affect one’s short-term cognitive function is worth further exploring to better understand the underlying mechanism of action.

In contrast to PC and lysoPC, urinary lysophosphatidylethanolamine (lysoPE) concentrations were significantly downregulated in the OH group. LysoPE are members of LPLs, which function as bioactive signaling lipids and metabolic intermediates in the cell membrane and are found to be associated with immune function and inflammatory conditions. Recent research evidence suggests that lysoPE may affect lipid accumulation and metabolism [27]. The serum lysoPE concentrations were significantly decreased in patients with nonalcoholic fatty liver compared with healthy controls [28]. An analysis of the urinary exposome of renal cancer patients also found lysoPE associated with a renal cell carcinoma signature [29]. However, the physiologic functions of lysoPE have not been fully illustrated.

In terms of amino acid metabolism, we found that the phenylalanine metabolism (0.50), the phenylalanine, tyrosine, and tryptophan biosynthesis (0.50), and the cysteine and methionine metabolism (0.10) had the most significant pathway impact. Phenylalanine is one of the essential amino acids and a member of the aromatic amino acids. Within the body, phenylalanine is the precursor of tyrosine, a crucial amino acid that is later converted into dopamine, serotonin, norepinephrine, and epinephrine. These important neurotransmitters and hormones in the sympathetic nerve system are irreplaceable regulators of multiple neurologic functions, including mood regulation and cognitive function. Melanin, which pigmentates the hair, skin, and eyes, is also synthesized from tyrosine. Defects in genes regulating phenylalanine metabolism can cause phenylketonuria, a severe condition that could lead to intellectual disability [30]. Moreover, due to the importance of tryptophan in the biosynthesis of serotonin, researchers proposed a possible mechanism for the onset and progression of depression that inflammation may alter tryptophan metabolism and decrease the bioavailability of tryptophan, resulting in a decreased serotonin concentration [31]. Correspondingly, previous studies suggest that fluid intake is associated with several mood states and psychologic conditions, including depression. An inverse correlation between plain water intake and depression was found among Iranian populations. Risk of depression for the lowest level of water consumption (<2 glasses per day) was almost twice as high as the reference group (≥5 glasses per day) (OR: 1.79; 95% CI: 1.32, 2.42; *P* < 0.0001) [32].

To summarize, the current study was a rare and innovative attempt to explore variations in urine metabolome and relevant metabolic pathways among subjects with different hydration states. We found that the urinary concentrations of various PLs and LPLs significantly differed between the optimal-hydrated and hypohydrated subjects. In addition, alterations in amino acid metabolism, including phenylalanine, tyrosine, tryptophan, cysteine, and methionine, were found. Metabolites and metabolic pathways mentioned above are involved in a wide range of renal, hepatic, cardiovascular, neuropsychologic, and metabolic functions. Therefore, further investigation is needed to establish a solid mechanism between the hydration state and health consequences.

## Author contributions

The authors’ responsibilities were as follows — GM: conceptualization and supervision; NZ and JZ: project administration; NZ, JZ, and YL: methodology; JZ, SL, and JL: investigation; YL and JL: data curation; YL: formal analysis and writing – original draft; GM, NZ, and JZ writing – review and editing; and all authors are involved in the manuscript revision and have read and agreed to the current version of the manuscript.

## Conflicts of Interest

All authors declare no conflict of interest.

## Funding

This research did not receive any specific grant from funding agencies in the public, commercial, or not-for-profit sectors.

## Data availability

Data described in the manuscript, code book, and analytic code will be available upon reasonable request.

## Appendix A Supplementary data

Multimedia component1 Multimedia component1
