# Supplementary material for: The association between hydration state and the metabolism of phospholipids and amino acids among young adults: a metabolomic analysis
Source: Curr Dev Nutr. 2024 Feb 1;8(3):102087. doi: 10.1016/j.cdnut.2024.102087 (PMC10904166; doi:10.1016/j.cdnut.2024.102087)
Supplement: Multimedia component1 [file mmc1.docx]

The Association Between Hydration States and Phospholipids and Amino Acids metabolism Among Young Adults: A Metabolomic Analysis

Yongwei Lin


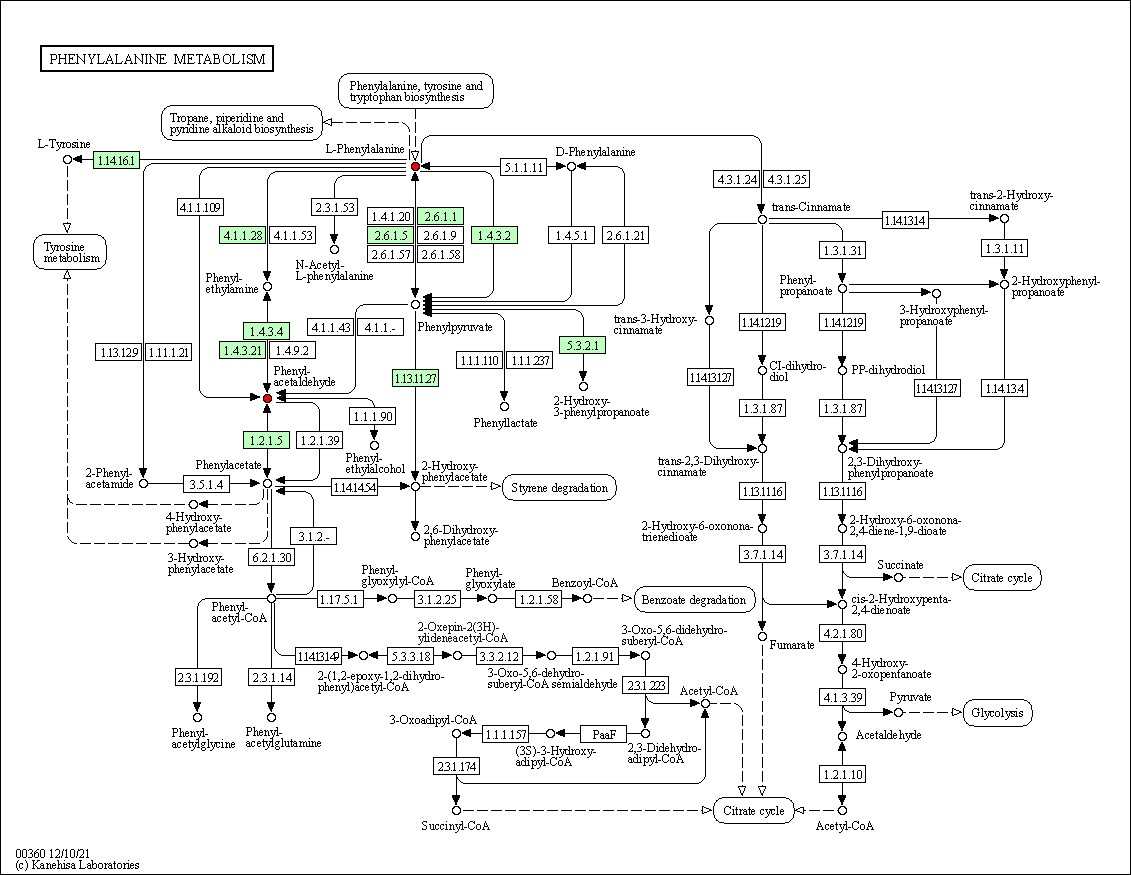


Table S1 The KEGG pathway maps of the phenylalanine metabolism with the affected pathway nodes labeled in red.


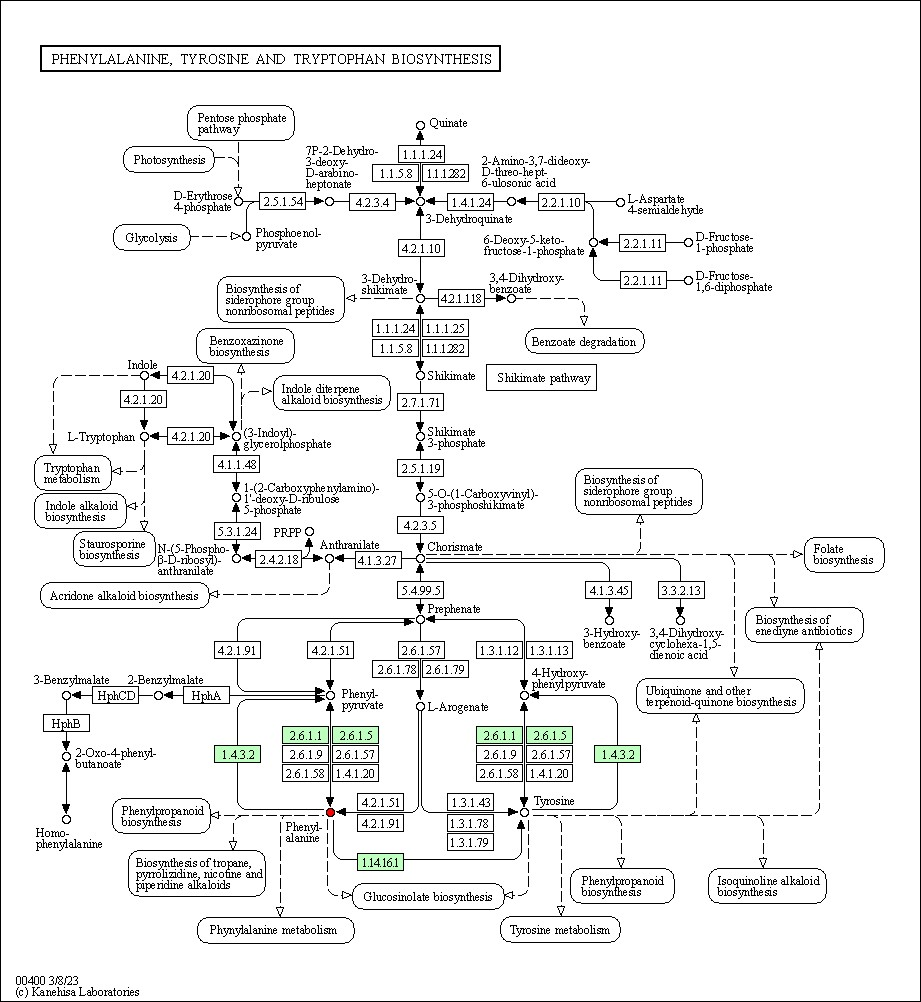


Table S2 The KEGG pathway maps of the phenylalanine, tyrosine, and tryptophan metabolism with the affected pathway nodes labeled in red


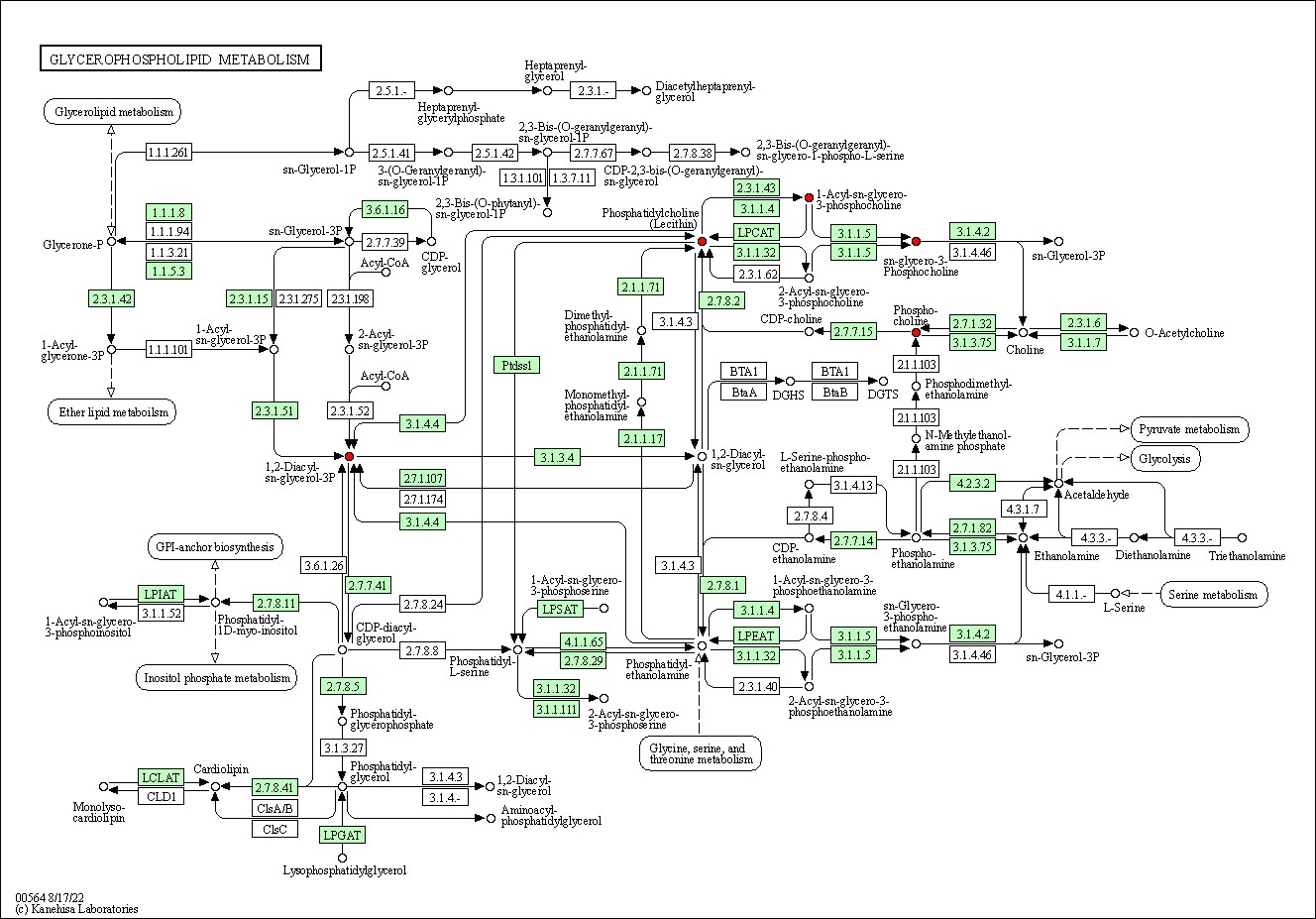


Table S3 The KEGG pathway maps of the glycerophospholipids metabolism with the affected pathway nodes labeled in red.
